# The autonomic cardiac nervous system and arrhythmogenesis: risk stratification in the foreseeable future

**DOI:** 10.1007/s00399-021-00777-0

**Published:** 2021-07-13

**Authors:** Heikki Huikuri

**Affiliations:** Oulu University Hospital and University of Oulu, Kajaanintie 50, 90220 Oulu, Finland

**Keywords:** Arrhythmias, Sudden cardiac death, Parasympathetic effects, Sympathetic effects, Electrocardiogram, Herzrhythmusstörungen, Plötzlicher Herztod, Parasympathische Wirkungen, Sympathische Wirkungen, Elektrokardiogramm

## Abstract

Both experimental and clinical studies have shown that the autonomic nervous system plays an important role in arrhythmogenesis. Many methods describing cardiovascular autonomic regulation have been developed and tested for use as predictors of arrhythmic and other cardiovascular events. The majority of studies have focused on patients with known cardiac disease, such as prior myocardial infarction or congestive heart failure. All-cause mortality, as well as non-sudden and sudden cardiac death have been used as main endpoints. Sudden cardiac death has often been considered to be equivalent to arrhythmic cardiac arrest. Despite promising results in this field, markers of the autonomic nervous system are still not routinely used in clinical practice, mainly due to the fact that measurement of these markers does not result in evidence-based therapeutic implications. There is still a lack of randomized trials using autonomic markers as pre-defined variables in selecting patients for the studies, which would have yielded results that an intervention reduces the arrhythmic or other endpoint in those with abnormal or impaired autonomic regulation. Hence, at present, the possible use of autonomic assessment in predicting life-threatening arrhythmias is restricted to individual cases at the borders of intervention guidelines.

## Abstract (de)

Experimentelle wie auch klinische Studien zeigen, dass das vegetative Nervensystem eine wichtige Rolle in der Entstehung von Herzrhythmusstörungen spielt. Es wurden viele Verfahren zur Beschreibung der kardiovaskulären vegetativen Regulation entwickelt und als Prädiktoren arrhythmischer und anderer kardiovaskulärer Ereignisse getestet. Die Mehrzahl der Studien konzentriert sich auf Patienten mit bekannter Herzerkrankung, etwa mit vorausgegangenem Myokardinfarkt oder Herzinsuffizienz. Die Gesamtmortalität wie auch der nichtplötzliche und plötzliche Herztod wurden als primäre Endpunkte herangezogen. Der plötzliche Herztod wird häufig mit dem arrhythmischen Herzstillstand gleichgesetzt. Trotz vielversprechender Ergebnisse in diesem Bereich werden Marker des vegetativen Nervensystems weiterhin nicht routinemäßig in der klinischen Praxis verwendet, hauptsächlich weil die Erfassung dieser Marker keine evidenzbasierten therapeutischen Konsequenzen hat. Es fehlen immer noch randomisierte Studien mit Markern der autonomen Regulation als vordefinierte Variablen für die Auswahl von Studienteilnehmern, die zeigen würden, dass eine Intervention die arrhythmiebezogenen oder andere Endpunkte bei Patienten mit abweichender oder gestörter vegetativer Regulation senkt. Folglich beschränkt sich die mögliche Nutzung einer Beurteilung der autonomen Regulation, um lebensbedrohende Arrhythmien vorherzusagen, gegenwärtig auf Einzelfälle im Grenzbereich von Therapieleitlinien.

## Background

For decades, abnormal cardiovascular autonomic regulation has been shown to be associated with an increased risk of all-cause and cardiac mortality, both sudden and non-sudden. Already in the early 1980s, animal studies by Schwartz et al. showed that impaired baroreflex sensitivity increases the risk of ischemia-induced ventricular fibrillation [1]. Subsequently, Kleiger et al. showed that measurement of heart rate variability from 24‑h Holter recording can identify post-infarction patients that had increased mortality during follow-up [2]. After these initial studies, a large number of investigations confirmed these seminal observations using various non-invasive indices able to quantify cardiovascular autonomic regulation [3]. Experimental and clinical data show that enhanced parasympathetic influence on the heart rate (HR) is generally antiarrhythmic, while increased sympathetic influence is generally pro-arrhythmic.

## Measures of the autonomic nervous system

HR variability from long-term electrocardiogram (ECG) recordings has been most commonly used in clinical studies to quantify cardiovascular autonomic regulation. HR variability has been applied in a vast number of studies [3]. Several different analysis methods have been used, such as time and frequency domain measures of HR variability, geometrical index, as well as measures based on fractals and non-linear analysis of HR dynamics. The focus has mainly been on risk stratification of patients after myocardial infarction or in those with congestive heart failure. Baroreflex sensitivity is another measure used in both experimental and clinical studies [1, 4]. This has been analyzed by using a phenylephrine test or HR turbulence in relation to ventricular ectopic beats, as well as by analyzing the so-called deceleration capacity of HR [5, 6]. All these measures mainly reflect the parasympathetic modulation of HR. More recently, a novel measure, periodic repolarization dynamics (PRD), has been introduced, which more closely reflects cardiac sympathetic regulation [7].

## Autonomic markers in risk stratification

The majority of studies assessing the prognostic power of autonomic markers have used all-cause mortality as the endpoint. Few studies have suggested that reduced or abnormal autonomic regulation may be specifically related to arrhythmic events and sudden cardiac death. The CARSIMA (Cardiac Arrhythmias and Risk Stratification after Myocardial Infarction) study showed that reduced very-low spectral component of HR variability, measured late after acute myocardial infarction, was the most powerful index among many other non-invasive risk markers in predicting the ventricular fibrillation or sustained ventricular tachycardia diagnosed by implantable arrhythmia devices [8]. A further sub-analysis of the CARSIMA study and another similar study (REFINE; Risk Estimation after Infarction, Noninvasive Evaluation) confirmed that HR variability and HR turbulence yield powerful prognostic information for arrhythmic events when measured late rather than early after acute myocardial infarction [9]. A recent large non-randomized European (EU-CERT; EUropean Comparative Effectiveness Research to assess the use of primary prophylacTic Implantable Cardioverter-Defibrillators) study showed that PRD predict mortality reduction associated with prophylactic implantable cardioverter defibrillators (ICD) in contemporarily treated patients with ischemic or non-ischemic cardiomyopathy [10]. Observational studies using various endpoints have shown that abnormal autonomic markers also predict non-sudden cardiac death. The largest study in this field showed that reduced HR variability/turbulence is in fact a stronger predictor of non-sudden than sudden cardiac death [11].

## Limitations of autonomic markers in risk stratification

Despite the large number of existing tests of autonomic nervous regulation, scant comparative data exist regarding their predictive value. No guidance exists as to which test or combination of autonomic markers is most appropriate in predicting arrhythmic events. Most observational studies have focused on post-infarction patients or on patients with congestive heart failure. It is not well understood how the autonomic markers provide prognostic information in the general population or in cardiac patients with preserved left ventricular function, who comprise the largest number of victims of sudden cardiac death.

Many observational studies in the field of autonomic markers in risk stratification also exhibit some statistical limitations. Although receiver operating characteristic (ROC) curves, Kaplan-Meier curves with log-rank tests, and Cox proportional hazards methods show significant *p*-values (< 0.05) of autonomic markers in the prediction of mortality and arrhythmic events, more rigorous statistical methods might be more persuasive. A statement from the American Heart Association recommends the use of C‑statistics and reclassification analysis criteria for the evaluation of risk markers [12]. A novel risk marker needs to improve risk prediction beyond established risk markers, that is, new markers should provide incremental prognostic information that could have clinical impact. As an example, one study showed that cardiorespiratory fitness was associated with risk of sudden cardiac death when Cox multivariate analyses were used. The categorized net reclassification index showed no change beyond established clinical risk markers, and if anything, trended in the wrong direction in the prediction of sudden cardiac death [13]. Since the function of the autonomic nervous system is closely related to many demographic and clinical markers, such as age, diabetes, hypertension, medications etc. [3], rigorous statistical methods should be used in observational study designs when analyzing the prognostic power of autonomic markers.

## Future directions

In view of the numerous studies that provide evidence for the power of clinical assessment by the measurement of cardiovascular autonomic function, it is reasonable to ask why these measurements are not widely used in clinical practice. Some of the limitations described above are the possible reasons for this. More importantly, there are no large randomized interventional studies or well-designed large observational registries that would provide prognostic evidence that measurement of autonomic function makes it possible to improve the prognosis of patients with impaired autonomic function. The limited use of autonomic function tests is also partially due to their complexity and abundance.

The large number of proposed tests and the inability to standardize the acquisition and analysis of parameters have contributed to preventing autonomic tests from becoming routinely used for the assessment of risk for life-threatening or fatal arrhythmias. The first step in this field is to obtain data from large comparative registries on which tests or combination of tests are most useful in the prediction of mortality and arrhythmic events. The predictive value of autonomic markers should also be compared to other risk markers, such as biomarkers obtained from blood samples, T‑wave alternans, and a number of other variables obtained from ECG recordings. In these attempts, rigorous statistical methods should be used to obtain information on how much autonomic and other risk markers really provide prognostic information beyond the demographic and traditional clinical data. The estimated level of risk should ideally be analyzed dynamically to determine whether and when diagnostic tests are appropriate. The prognostic value of autonomic markers should also be tested in various populations, such as cardiac patients with and without depressed left ventricular function, as well as in general population samples. Finally, randomized interventional trials should be started by using an easily obtainable autonomic marker as a predefined variable alone or in combination with some other predefined variables for inclusion in the trial. A randomized ICD trial is perhaps most appropriate for those with a prior cardiac event and severely or modestly depressed left ventricular function. An optimized medical treatment and/or revascularization trial might be appropriate for cardiac patients with mildly depressed left ventricular function, and finally, a wide-scale life-style intervention trial, including exercise and diet counseling, should be performed for subjects without known heart disease but impaired cardiac autonomic function.

## Declarations

For this article no studies with human participants or animals were performed by any of the authors. All studies performed were in accordance with the ethical standards indicated in each case.
